# Levels and correlates of physical activity and capacity among HIV-infected compared to HIV-uninfected individuals

**DOI:** 10.1371/journal.pone.0262298

**Published:** 2022-01-21

**Authors:** Brenda Kitilya, George PrayGod, Robert Peck, John Changalucha, Kidola Jeremiah, Bazil Baltazar Kavishe, Henrik Friis, Suzanne Filteau, Daniel Faurholt-Jepsen, Rikke Krogh-Madsen, Soren Brage, Mette F. Olsen

**Affiliations:** 1 Mwanza Research Centre, National Institute for Medical Research, Mwanza, Tanzania; 2 Weill Bugando School of Medicine, Mwanza, Tanzania; 3 Weill Cornell Medicine, New York, New York, United States of America; 4 Department of Nutrition, Exercise and Sports, University of Copenhagen, Copenhagen, Denmark; 5 Department of Population Health, London School of Hygiene and Tropical Medicine, London, United Kingdom; 6 Department of Infectious Diseases, Rigshospitalet, Copenhagen, Denmark; 7 Centre for Physical Activity Research, Rigshospitalet, University of Copenhagen, Copenhagen, Denmark; 8 Department of Infectious Diseases, Copenhagen University Hospital, Hvidovre, Copenhagen, Denmark; 9 MRC Epidemiology Unit, University of Cambridge, Cambridge, United Kingdom; University of Cape Town Faculty of Science, SOUTH AFRICA

## Abstract

**Introduction:**

In the HIV-infected individuals, physical activity improves physical strength, quality of life and reduces the risk of developing non-communicable diseases. In Sub-Saharan Africa, HIV-infected patients report being less active compared to HIV-uninfected individuals. We assessed the levels and correlates of objectively measured physical activity and capacity among HIV-infected antiretroviral therapy (ART)-naive individuals compared to HIV-uninfected individuals in Mwanza, Tanzania.

**Method:**

We conducted a cross-sectional study among newly diagnosed HIV-infected ART-naive individuals and HIV-uninfected individuals frequency-matched for age and sex. Socio-demographic data, anthropometrics, CD4 counts, haemoglobin level, and C-reactive protein (CRP) were collected. Physical activity energy expenditure (PAEE) was assessed as measure of physical activity whereas sleeping heart rate (SHR) and grip strength were assessed as measures of physical capacity. Multivariable linear regression was used to assess the correlates associated with physical activity and capacity.

**Results:**

A total of 272 HIV-infected and 119 HIV-uninfected individuals, mean age 39 years and 60% women participated in the study. Compared to HIV-uninfected individuals, HIV-infected had poorer physical activity and capacity: lower PAEE (-7.3 kj/kg/day, 95% CI: -11.2, -3.3), elevated SHR (7.7 beats/min, 95%CI: 10.1, 5.3) and reduced grip strength (-4.7 kg, 95%CI: -6.8, -2.8). In HIV-infected individuals, low body mass index, moderate-severe anaemia, low CD4 counts and high CRP were associated with lower physical activity and capacity. In HIV-uninfected individuals, abdominal obesity and moderate anaemia were associated with lower physical activity and capacity.

**Conclusion:**

HIV-infected participants had lower levels of physical activity and capacity than HIV-uninfected participants. Correlates of physical activity and capacity differed by HIV status. Management of HIV and related conditions needs to be provided effectively in health care facilities. Interventions promoting physical activity in these populations will be of importance to improve their health and reduce the risk of non-communicable diseases.

## Introduction

Physical activity improves physical strength and capacity [1], and reduces the risk of non-communicable diseases (NCDs) and mortality [2,3]. In HIV-infected individuals, physical activity also improves well-being, has positive psychological effects and may reduce the side effects of antiretroviral therapy (ART) such as anxiety, fatigue, muscle pains and headaches [4]. However, emerging evidence suggests that HIV-infected have lower physical activity and capacity, including reduced physical strength [5,6] than HIV-uninfected individuals which may lead to poor quality of life and increased risks of developing NCDs.

Most of the data on physical activity and capacity among HIV-infected individuals come from high-income countries [7,8] and studies in Sub-Saharan Africa (SSA) are few with conflicting results [9,10]. Such conflicting results could be explained by differences in participants’ demographic characteristics, stages of HIV-infection or measures used to determine physical activity. In SSA, the commonly used measure to assess physical activity has been self-report [11,12], which is subject to recall bias and thus potentially compromising validity of results [13,14]. Objective measures of physical activity have been shown to be more precise compared to subjective measures [15] because, they provide a more continuous evaluation of free-living activity [16,17] and capture better, the intricacies of physical activity dimensions [18]. Specifically, using combined heart rate and accelerometer monitors with biomarkers of physical activity and capacity, physical activity energy expenditure (PAEE) and sleeping heart rate (SHR), and additional use of hand grip strength in research, may improve the assessment of overall habitual physical activity among individuals [13,19–21]. Thus, this will further provide an in-depth understanding of their physical capacity, and thus contribute to empirical evidence.

A previous study in Tanzania which assessed physical activity using similar objective measures among tuberculosis (TB) patients found HIV co-infection was associated with lower levels of physical activity [22]. Besides the Tanzanian study, a recent systematic review found that, there were only two other small studies in Africa which had assessed physical activity among HIV-infected individuals using objective measures [23]. Both of these studies reported that advanced stages of HIV infection were associated with lower levels of physical activity [23,24]. This dearth of high-quality physical activity data from HIV-infected individuals limits development of strategies to improve physical activity and thereby general health and well-being of HIV-infected individuals in Africa. Furthermore, apart from a limited number of studies using objective measures of physical activity, studies assessing the role of HIV infection and other correlates affecting physical activity levels in HIV-infected individuals by comparing with individuals who are not HIV-infected are scarce in the SSA [23]. This study aimed to assess levels and correlates of objectively measured physical activity and capacity among newly diagnosed HIV-infected ART-naive compared to HIV-uninfected individuals in Tanzania.

## Method

### Study design and setting

This was a cross–sectional study conducted from February 2017 to February 2018 in Mwanza, Tanzania, where the prevalence of HIV infection was 7.2% [25]. The study was embedded within the Chronic Infection, Co-morbidities and Diabetes in Africa (CICADA) study, registered at http://clinicaltrials.gov as NCT03106480. The CICADA study design and prevalence of diabetes in the cohort has been published [26]. Participants who were attending CICADA study visits were invited to participate in this study.

### Sample size

We aimed to have a sample size with adequate power to demonstrate the difference in physical activity and capacity between HIV-infected ART-naïve and HIV-uninfected individuals. Using data available from the CICADA cohort: 272 HIV-infected and 119 HIV-uninfected individuals allowed us to detect an expected difference in physical capacity indicated by grip strength of 4.2 kg (SD 6.7) [27] at significance 0.05 level and power of 0.8.

### Recruitment of participants

HIV-infected participants were recruited if aged ≥18 years, newly diagnosed HIV-infected ART-naïve willing to start ART immediately after study enrolment, not pregnant, and residing in Mwanza. The current Tanzanian HIV policy and the national multi-sectoral strategic framework for HIV and AIDS adopted Test and Treat policy from WHO in 2016 [25,28]. Hence, HIV-infected participants were recruited after testing, assessed before they started ART at the ART clinics.

HIV-uninfected individuals were recruited from the same communities as the HIV-infected ART-naive and were frequency-matched for age and sex at a ratio of 2:1. Communities were defined as streets or sub-villages with a minimum of 200 households as per administrative setup of the local government. With the support of the respective community leaders, we obtained a list of households in the community. We then randomly selected three households from which an eligible individual (not severely sick, not having any HIV clinical symptoms) was identified. In case of refusal to participate, we repeated the process until suitable participants were found. To confirm HIV-negative status, participants in the comparison group were tested for HIV and if found to be HIV-infected, they were invited to participate in the HIV-infected group and were referred for treatment.

### Data collection procedures

#### Questionnaire data

Information on demographics, socio-economic status, level of education, marital status, and occupation were collected by a trained research staff using electronic structured questionnaire in a CSpro version 6.3 data capturing system (CSPro 6.3, Census bureau, USA).

#### Anthropometric measurements

Trained researchers collected anthropometric measurements in triplicate using standardized methods and the median of the three recordings was used for analysis. With minimal clothing and while barefoot, participant’s body weight was measured to the nearest 0.1 kg using a digital scale (SECA, 877, Hamburg, Germany). Height was measured to the nearest 0.1 cm using a wall fitted stadiometer (SECA, FS317, Germany). Waist circumference was measured at the midpoint between the lower part of the last rib and the top of the hip bone using a tape measure.

#### Physical activity and capacity

PAEE (kj/kg/day), as an indicator of habitual physical activity, and SHR (beats/min) as an indicator of physical aerobic capacity, were assessed during daily living using a combined heart rate and accelerometer (Actiheart, Camtech, Cambridge UK) [29,]. The device was fitted using two electrocardiogram (ECG) electrodes placed one on the upper left side of the chest and another one laterally on top of the chest. Participants performed a step test for individual calibration of relationship between heart rate and energy expenditure as described elsewhere [30]. The step test was done at the study clinic using a step board with a height of 20 cm for 5 minutes followed by a 2-minute recovery period. Participants were then set up with the monitor to be worn for five days to assess habitual PAEE and SHR. Participants were informed of the purpose of wearing the monitor and were requested to ensure they wore it at all times while continuing with their day-to-day activities except they were not to wear it when performing deep-water activities. On the third day, participants were requested to return to the clinic for change of ECG electrodes and device monitoring. If at any point the data had recording errors, participants were requested to extend the number of days from five to ten days of monitoring.

Data on activity level, SHR and heart rate (heart beats per minute) were retrieved from the monitor and were processed using Gaussian robust regression model to remove noisy data [31]. Observations with total recording time of less than 72 hours over the five days, noisy data on heart rate and/or acceleration were removed. Sleeping heart rate was determined from all the days with completed 24 hrs measurements. Acceleration and heart rate collected from the monitor were combined using branched equations to estimate daily PAEE [16]. Participants who were not able to complete a step test for individual calibration of their heart rate-energy expenditure relationship, we used a group calibration equation which was derived as the average age and sex-specific calibration curves from the participants who performed the step test.

Grip strength as an indicator of the muscular strength, aspect of physical capacity was measured to the nearest 0.1 kg using a digital grip strength dynamometer (Takei Scientific Instruments, Tokyo, Japan). Four measurements were taken and the mean of the two highest measurements (one from each hand) was used for analysis.

#### HIV, CD4, and C-reactive protein testing

A whole venous blood sample was collected, processed and used for HIV, CD4 counts, hemoglobin and C-reactive protein (CRP) testing. HIV-infection was diagnosed based on two rapid tests: Bioline (SD Bioline Standard Diagnostic Inc, Republic of Korea) and Unigold (Unigold Trinity Biotech, IDA Business Park, Bray Co, Wicklow, Ireland). Discordant samples were tested using ELISA (11 Vironostika-HIV Ag/Ab Micro Elisa systems, Biomerieuxbv, Netherlands). CD4 counts (cells/uL) were determined using Cyflow Partech machine (Partech GmbH, Munster, Germany). Hemoglobin level was measured using haematology analyser (Coulter, Model Act5 diff AL, Beckman Coulter Inc, USA). CRP was measured using sandwich ELISA [32].

### Data management and statistics

Data were processed and analysed using Stata version 13 (College Station, Texas, USA). CD4 counts were categorised as ≤200, 201–499 and ≥500 (cells/uL), based on WHO cut-off points, anaemia was classified as no anaemia (men: ≥13g/dL; women: ≥12g/dL), mild (women: 11–11.9 g/dL; men: 11–12.9 g/dL), moderate (8.0–10.9 g/dL, both men and women) and severe anaemia (<8.0 g/dL, both women and men) [33], body mass index (BMI) was calculated as weight (kg)/height (m)^2^ and categorised as underweight (<18.5 kg/m^2^), normal weight (18.5–24.99 kg/m^2^) and overweight (≥25.0 kg/m^2^). Waist circumference (cm) was defined as normal (women: ≤88 cm; men: ≤102 cm) and abdominal obesity (women: >88 cm; men: >102 cm) [34].

All correlates were tested for collinearity with variance inflation factors. No corrective measures were required since all correlates had variance inflation factors <5. Normality of continuous variables was checked using histograms. Log transformation was done to achieve normal distribution for CRP. Participants’ characteristics were summarised as number (percentages), median (IQR), and mean (SD) stratified by HIV-status. For comparison of two means by HIV-status, chi-square tests were used for categorical variables and t-tests were used for continuous variables. Levels of PAEE, SHR, and grip strength were stratified by HIV status and linearly regressed to the assessed correlates: age, sex, education, employment, BMI, waist circumference, haemoglobin levels, CD4 count, and log CRP. In multivariable analysis, the associations between the potential correlates and PAEE, SHR, and grip strength, respectively, were adjusted for age and sex. A test of interaction was included in the adjusted model to assess whether associations with correlates differed significantly between HIV-infected and HIV-uninfected individuals. The correlations were presented as beta coefficients with 95% confidence interval (CI) in HIV-infected and HIV-uninfected individuals, the difference of the beta coefficients, 95%CI of the HIV-infected versus HIV-uninfected individuals and interaction p-value respectively. A p-value <0.05 was regarded as statistically significant.

### Ethics consideration

This study was conducted in accordance with the Helsinki declaration of 1964. Ethical approval for the study was provided by the Medical Research Coordinating Committee of the National Institute for Medical Research and the Catholic University of Health and Allied Sciences Ethics Review Board in Tanzania. All eligible participants were informed of the study purpose, procedures and provided oral and written informed consent prior to their enrolment into the study.

## Results

A total of 457 HIV-infected ART-naive and HIV-uninfected individuals participated in this study. After data processing, 66 (14%) participants were removed from the analysis due to missing data either on acceleration or heart rate or both, leaving 391 (86%) participants for the present analysis with 259 (66%) having completed the step test among HIV-infected and HIV-uninfected individuals. The average recording length of physical activity data was 4.3 days.

Participants’ baseline characteristics are displayed in Table 1. There were 272 HIV-infected ART-naive and 119 HIV-uninfected individuals. Mean age and proportion of women were similar in the two groups. The majority of participants had primary level of education and worked in the informal sector as self-employed/petty traders.

**Table 1:** Background characteristics of 391 participants by HIV status.

	HIV-infected (n = 272)	HIV-uninfected (n = 119)	P values
Female sex, n (%)	163 (60.0)	72 (61.0)	0.92
Age (years), mean (SD)^a^	38.5 (11.0)	39.8 (10.5)	0.27
Education level^b^, n (%)			
Never went to school	51 (18.9)	5 (4.2)	<0.01
Primary school	186 (68.9)	84 (70.6)	
Higher education/vocational	33 (12.2)	30 (25.2)	
Employment^b^, n (%)			
Salary employee	50 (18.5)	12 (10.1)	0.09
Self-employed /other	182 (67.4)	86 (72.3)	
Unemployed/housewife	38 (14.1)	21 (17.9)	
Body mass index^b^ (kg/m^2^), mean (SD)^a^	20.84 (3.9)	24.33 (4.9)	<0.01
Body mass index (kg/m^2^), n (%)			
Normal (18.5—<25)	156 (57.3)	70 (59.0)	<0.01
Underweight (<18.5)	72 (26.5)	6 (5.0)	
Overweight (>25)	44 (16.1)	43 (36.1)	
Abdominal obesity^c^(cm), n (%)	22 (8.0)	29 (24.4)	
Hemoglobin (g/dL), mean (SD)^a^	11.4 (2.8)	13.5 (2.2)	<0.01
Anaemia, n (%)			
No anaemia^d^	102 (38.0)	95 (79.8)	<0.01
Mild^e^	64 (23.5)	14 (11.7)	
Moderate^f^	88 (32.4)	7.(5.9)	
Severe^g^	18 (6.6)	3 (2.5)	
CD4 counts^b^ (cells/μL), n (%)			
≥500	36 (13.2)	97 (81.5)	<0.01
201–499	100 (36.8)	20 (16.8)	
≤ 200	136 (50.0)	2 (1.7)	
CRP^b^^,^ ^i^ (mg/L), median, (IQR)^h^	3.43 (1.2, 17.1)	1.45 (0.7, 3.4)	0.01
Physical activity energy expenditure (kj/kg/day), mean, (SD)^a^	33.4 (18.8)	40.7 (17.0)	<0.001
Sleeping heart rate (beats/min), mean, (SD)^a^	68.5 (12.1)	60.8 (7.9)	<0.001
Grip strength (Kg), mean (SD)^a^	26.4 (8.6)	31.3 (9.8)	<0.001

^a^ Standard deviation;

^b^ Missing data: Two on education, 10 on body mass index, three on CRP, four on CD4 count;

^c^ waist circumference > 102 for men or > 88 for women;

^d^ no anaemia ≥12.0 g/dL for men/≥13 g/dL for women;

^e^ mild anaemia 11.0–11.9 g/dL for men/11.0–12.9 g/dL women;

^f^ moderate anaemia 8.0–10.9 g/dL for men/8.0–10.9 g/dL for women;

^g^ severe anemia <8.0 g/dL for men/<8.0 g/dL for women;

^h^ interquartile range (IQR)

^i^C-creative protein.

P-values were obtained from t-test for continuous variable or X^2^ test for categorical variable.

Two-thirds of the participants had normal BMI but there was a higher proportion of underweight among HIV-infected (27% vs 5%) and a higher proportion of overweight (36% vs 16%) and abdominal obesity (24% vs 8%) among HIV-uninfected individuals. Most of HIV-uninfected individuals had normal levels of haemoglobin (80%) whereas specifically moderate anaemia was common among HIV-infected individuals (32%). CRP levels were higher in HIV-infected than in HIV-uninfected individuals.

HIV-infected had lower physical activity, PAEE (-7.3 kj/kg/day, 95%CI: -11.2, -3.3) compared to HIV-uninfected individuals. Likewise, SHR was higher (7.7 beats/min, 95%CI: 10.1, 5.3) and grip strength was lower (-4.7 kg, 95%CI: -6.7, -2.8) implying poor physical capacity in HIV-infected compared to HIV-uninfected individuals.

Table 2 displays correlates of physical activity energy expenditure. Among HIV-infected low BMI (-5.4 kj/kg/day, 95%CI: -10.4, -0.4), moderate anaemia (-13.8 kj/kg/day, 95%CI: -18.8, -8.8), CD4 counts ≤200cells/uL (-9.9 kj/kg/day, 95%CI: -16.5, -3.3) and log CRP (-9.7 kj/kg/day, 95%CI: -12.4,-7.0) were associated with lower PAEE.

**Table 2:** Correlates of physical activity energy expenditure (kj/kg/day) among HIV-infected and HIV-uninfected individuals.

	HIV-infected (n = 272)	HIV-uninfected (n = 119)	HIV-infected versus HIV-uninfected	
	β (95%CI)^a^	β (95%CI)^a^	β difference (95%CI)^a^	Interaction p value
Body mass index^b^ (kg/m^2^)				
Underweight	-5.4 (-10.4, -0.4)	-1.7 (-16.5, 13.1)	-3.7 (-19.4, 11.9)	
Normal	Ref	Ref	Ref	0.14
Overweight	3.2 (-2.8, 9.2)	-5.0 (-11.7, 1.8)	8.2 (-0.8, 17.1)	
Waist circumference (cm)				
Normal	Ref	Ref	Ref	0.06
Abdominal obesity^c^	-1.2 (-9.1, 6.7)	-11.4 (-19.1, -3.6)	10.1 (-0.6, 20.8)	
Anaemia				
No-anaemia^d^	Ref	Ref	Ref	**<0.001**
Mild^e^	-5.2 (-10.6, 0.2)	1.3 (-8.5, 11.03)	-6.5 (-17.7, 4.7)	
Moderate^f^	-13.8 (-18.8, -8.8)	13.7 (0.5, 26.9)	-27.4 (-41.4, -13.4)	
Severe^g^	-13.8 (-22.5, -5.2)	-12.0 (-31.7, 7.8)	-1.9 (-23.3, 19.6)	
CD4 counts^b^ (cells/μL)				
≥500	Ref	Ref	Ref	**0.03**
201–499	-2.9 (-9.6, 3.9)	-1.2 (-9.6, 7.3)	-1.7 (-12.5, 9.2)	
≤200	-9.9 (-16.5, -3.3)	24.0 (-0.7, 48.6)	-33.9 (-59.3, -8.4)	
Log CRP^b^^,^ ^h^(mg/L)	-9.7 (-12.4, -7.0)	-2.7 (-9.3, 3.9)	-7.0 (-14.1, 0.1)	**0.05**

^a^ Confidence interval;

^b^ Missing data:10 on body mass index, four on CRP, four on CD4 count;

^c^ waist circumference > 102 for men or > 88 for women;

^d^ no anaemia ≥12.0 g/dL for men/≥13 g/dL for women;

^e^ mild anaemia 11.0–11.9 g/dL for men/11.0–12.9 g/dL women;

^f^ moderated anaemia 8.0–10.9 g/dL for men/8.0–10.9 g/dL for women;

^g^ severe anaemia <8.0g/dL for men/<8.0 g/dL for women;

^h^ C-creative protein.

Abdominal obesity (-11.4 kj/kg/day, 95%CI: -19.1, -3.6) was the only correlate associated with lower PAEE among HIV-uninfected individuals. The tests for interaction showed that HIV-status modified the relationship between all the assessed correlates and PAEE (p≤0.05) except for abdominal obesity and BMI.

Low BMI and CD4 counts ≤200 cells/uL were associated with elevated SHR (6.8 beats/min, 95%CI: 3.9, 9.8) and (10.6 beats/min, 95%CI: 6.8, 14.5) respectively in HIV-infected, while they were not associated in HIV-uninfected individuals. The interaction tests were not significant (p = 0.16 and p = 0.28) respectively. Moderate anaemia and log CRP were associated with elevated SHR (10.3beats/min, 95%CI: 7.3, 13.5 and 7.0 beats/min, 95% CI: 5.5, 8.6) in HIV-infected only (interaction p = 0.04 and p<0.001). Abdominal obesity was not associated with SHR in either group (Table 3).

**Table 3:** Correlates of sleeping heart rate (beats/min) among HIV-infected and HIV-uninfected individuals.

	HIV-infected (n = 272)	HIV-uninfected (n = 119)	HIV-infected versus HIV-uninfected	
	β (95%CI)^a^	β (95%CI)^a^	β difference (95%CI)^a^	Interaction p value
Body mass index^b^ (kg/m^2^)				
Underweight	6.8 (3.9, 9.8)	0.5 (-8.3, 9.3)	6.3 (-3.0, 15.6)	
Normal	Ref	Ref	Ref	0.16
Overweight	-0.8 (-4.4, 2.8)	2.1 (-1.9, 6.2)	-3.0 (-8.3, 2.4)	
Waist circumference^c^ (cm)				
Normal	Ref	Ref	Ref	0.17
Abdominal obesity	-2.7 (7.6, 2.1)	1.8 (-3.0, 6.5)	4.5 (-11.0, 2.0)	
Anaemia				
No anaemia^d^	Ref	Ref	Ref	0.04
Mild^e^	1.4 (-1.8, 4.7)	-1.9 (-7.7, 3.9)	-3.4 (-3.30, 10.0)	
Moderate^f^	10.3 (7.3, 13.5)	-0.4 (-8.3, 7.4)	10.7 (2.4, 19.0)	
Severe^g^	6.6 (1.5, 11.8)	-2.3 (14.1, 9.4)	9.0 (-3.8, 21.74)	
CD4 Counts^b^ (cells/μL)				
≥500	Ref	Ref	Ref	0.28
201–499	4.5 (0.5, 8.5)	3.6 (-1.3, 8.6)	0.9 (-5.5, 7.2)	
≤200	10.6 (6.8, 14.5)	-1.3 (-15.8, 13.1)	12.0 (-3, 26.9)	
Log CRP^b^^,^ ^h^ (mg/L)	7.0 (5.5, 8.6)	0.9 (-2.9, 4.8)	6.11 (2.0, 10.3)	**<0.01**

^a^ Confidence interval;

^b^ Missing data:10 on body mass index, four on CRP, four on CD4 count;

^c^ waist circumference > 102 for men or > 88 for women;

^d^ no anaemia ≥12.0 g/dL for men/≥13 g/dL for women;

^e^ mild anaemia 11.0–11.9 g/dL for men/11.0–12.9 g/dL women;

^f^ moderate anaemia 8.0–10.9 g/dL for men/8.0–10.9 g/dL for women;

^g^ severe anaemia <8.0 g/dL for men/<8.0 g/dL for women;

^h^ C-creative protein.

Table 4 displays the correlates of grip strength. Low BMI (-5.6 kg, 95%CI: -7.4, -3.8), moderate anaemia (-3.9 kg, 95%CI: -5.8, -2.0), and low CD4 (-2.9 kg, 95%CI: -5.4, -0.4) were associated with lower grip strength in HIV-infected, while only moderate anaemia was associated with lower grip strength (-5.1 kg, 95%CI: -10.2, -0.1) in HIV-uninfected individuals, but tests of interaction were not significant (p>0.10). In addition, log CRP was associated with lower grip strength (-3.3 kg, 95%CI: -4.3, -2.3) in HIV-infected individuals only (interaction p = <0.002). Abdominal obesity was not associated with grip strength in either of the group.

**Table 4:** Correlates of grip strength (kg) among HIV-infected and HIV-uninfected individuals.

	HIV-infected (n = 272)	HIV-uninfected (n = 119)	HIV-infected versus HIV-uninfected	
	β (95%CI)^a^	β (95%CI)^a^	β difference (95%CI)^a^	Interaction p value
Body mass index^b^ (kg/m^2^)				
Underweight	-5.6 (-7.4–3.8)	-0.9 (-6.2, 4.4)	-4.7 (-10.3, 0.9)	
Normal	Ref	Ref	Ref	0.23
Overweight	1.1 (-1.0, 3.3)	0.9 (-1.6, 3.3)	0.3 (-3.0, 3.5)	
Waist circumference(cm)				
Normal	Ref	Ref	Ref	0.26
Abdominal obesity^c^	2.0 (-1.0, 5.0)	-0.3 (-3.2, 2.7)	2.3 (-1.8, 6.4)	
Anaemia				
No anaemia^d^	Ref	Ref	Ref	0.69
Mild^f^	-1.4 (-3.4, 0.7)	-1.8 (-5.6, 1.9)	0.5 (-3.8, 4.7)	
Moderate^g^	-3.9 (-5.8, -2.0)	-5.1 (-10.2, -0.1)	1.2 (-4.1, 6.6)	
Severe^h^	-2.5 (-5.8, 0.7)	-7.3 (-14.8, 0.3)	4.8 (-3.4, 13.0)	
CD4 Counts^b^ (cells/μL)				
≥500	Ref	Ref	Ref	0.53
201–499	-0.2 (-2.8, 2.3)	-0.6 (-3.8, 2.6)	0.4 (-3.7, 4.4)	
≤200	-2.9 (-5.4, -0.4)	2.3 (-7.0, 11.6)	-5.2 (-14.8, 4.4)	
Log CRP^b^^,^ ^h^ (mg/L)	-3.3 (-4.3, -2.3)	1.0 (-1.5, 3.6)	-4.4 (-7.1, -1.7)	**0.002**

^a^ Confidence interval;

^b^ Missing data:10 on body mass index, four on CRP, four on CD4 count;

^c^ waist circumference > 102 for men or > 88 for women;

^d^ no anaemia ≥12.0 g/dL for men/≥13 g/dL for women;

^e^ mild anaemia 11.0–11.9 g/dL for men/11.0–12.9 g/dL;

^f^ moderate anaemia 8.0–10.9 g/dL for men/8.0–10.9 g/dL for women;

^g^ severe anaemia <8.0g/dL for men/<8.0 g/dL for women;

^h^ C-creative protein.

## Discussion

Using objective measures, this study showed newly diagnosed HIV-infected ART-naïve individuals have lower levels of physical activity and capacity compared to HIV-uninfected individuals. The main factors correlated to lower physical activity and capacity in HIV-infected were low BMI, anaemia, low CD4 counts as a marker of disease progression, and increased CRP as a marker of inflammation. Anaemia and abdominal obesity were the only correlates associated with reduced physical activity and capacity in HIV-uninfected individuals.

The results found on levels of physical activity indicated by reduced PAEE in HIV-infected, complement results from other studies conducted in high-income countries showing that, HIV-infected have reduced levels of physical activity which is ranging from 19–73% due to HIV-inflammation, opportunistic infections, anaemia, metabolic complications [35,36] and under-nutrition [37]. Similar results were observed in two studies conducted in Malawi and Ethiopia, where HIV-infected individuals who were in advanced stages of the HIV disease and anaemic had lower levels of physical activity and capacity [27,38]. Based on these findings, in the SSA, interventions promoting physical activity at early stages of the HIV- infection are urgently needed to improve muscle strength, quality of life as well as general well being.

The present study also highlighted the associations between anaemia and physical activity and capacity among HIV-infected and HIV-uninfected individuals. The prevalence of anemia in the HIV population in other studies was found to be even higher compared to uninfected population, and it ranged from 45%-70 [39]. Anemia in HIV infected may be caused by opportunistic infections including TB, poor absorption of iron from the diet and the HIV-inflammation on the bone marrow which may result in dysregulation of erythropoiesis resulting in anaemia [39]. The mechanism of reduced physical capacity and activity from anemia is mainly a result of having reduced haemoglobin levels, decreased oxygen transportation to skeletal muscle leading to impaired physical performance [40,41]. Nevertheless, our findings of an association between anaemia and lower physical capacity indicate that there is a need for effective screening and management of anaemia, not only in HIV-infected but also in HIV-uninfected individuals, to reduce inability to do physical activity, which eventually may lead to poor quality of life, a risk of NCDs and poor economic productivity at the individual level.

In addition, we found that HIV-uninfected individuals with abdominal obesity had reduced physical activity potentially increasing the risk of NCDs. The prevalence of metabolic syndrome has been rising in the SSA over the years. A 2019 review reported that among HIV-uninfected individuals, the prevalence was about 12% [42]. In Tanzania, the prevalence of abdominal obesity is 25% and higher in women with an estimate of 35% [43]. Our study results on abdominal obesity suggest that there will be higher burden of diabetes and cardiovascular diseases in this population in the near future. These findings call for a need of aggressive interventions to promote physical activity in the general population.

HIV-associated wasting syndrome is characterised by involuntary loss of body lean mass and fat mass, a case definition of HIV-infection [44], resulted from several factors including, reduced food intake, malabsorption of nutrients, reduced utilization of nutrients and increased energy expenditure especially during opportunistic infections [45]. Weight loss in HIV-infected is accompanied by muscle fatigue and weakness which explains the reduced grip strength [46]. Grip strength not only is an indicator of lower physical capacity but also a marker of frailty, disability and nutritional deficiency [24]. In the present study, HIV-infection and HIV-related conditions such as low BMI was associated with reduced grip strength. These results complement a systematic review and meta–analysis data which reported HIV-infected patients have reduced grip strength by 4 kg [47], lower physical function and higher risk of frailty compared to HIV-uninfected individuals [5]. Thus, HIV- infected patients should be encouraged to do regular physical activity to help re-gain muscle strength and improve physical functioning.

Higher SHR is a predictor of cardiovascular diseases and all-cause mortality [48]. The current study found that, HIV-infected individuals had high SHR compared HIV-uninfected individuals especially those with moderate or severe anaemia, low BMI, and low CD4 counts, indicating that HIV-infected patients are at a higher risk of NCDs.

In SSA, previous studies on physical activity and HIV-infection mainly used subjective measures of physical activity which may have contributed to inconsistent results [23,49]. In particular, self-report methods are prone to recall bias [14] compared to objective measures. The objective measure, combined heart rate and accelerometer monitor directly assesses more than one dimensions of physical activity and has the ability to capture a variety of metrics including minutes and intensity of activity, bouts of activity and heart beats [13,16,50] which are the major biological outputs in the calculations of PAEE, and among the known appraised metrics to indicate physical activity [13]. Furthermore, validation studies of PAEE assessed using accelerometers and referenced using doubly labelled water as a gold standard from Europe and Africa have considered the PAEE estimate from combined heart rate and movement sensing provides valid data in assessing physical activity levels at group level research [51]. Thus, the use of objective measure, PAEE in this study marks the new era of improving validity of physical activity data in the SSA.

### Strengths and limitations

The strengths of this study include the use of an objective measure to assess physical activity and capacity using PAEE, and SHR parameters in both HIV-infected and HIV-uninfected individuals. An additional strength is that, we used individual calibration of heart rate via a step test in the majority of the sample and, based on this data, developed a population-specific group calibration equation for estimating PAEE for use in those who did not complete a step test. Nonetheless, participants without a step test were more likely to have more advanced HIV disease and therefore also more likely to be less fit, the result of which would be a small residual positive bias of their PAEE estimates. For this reason, the difference in physical activity levels between HIV-infected and HIV-uninfected individuals may in fact be greater than we report here. Future studies should consider offering alternative exercise testing such as self-paced walk tests for those unable to complete tests with prescribed exercise loads. Further, the present findings add to the literature by comparing the role of HIV-infection and correlates on physical activity, and capacity in HIV-infected compared to HIV-uninfected individuals in a large sample in the SSA.

The main limitations for this study were participants’ low compliance with wearing the combined monitors for specific time periods like during sleep or due to allergic skin reactions to the ECG electrodes. In addition, in this study, we could not establish the time or duration of the infection of HIV-infected individuals, which may be of importance to our findings. Furthermore, this study was a cross-sectional, limiting causal inference of the relationship between the correlates and physical activity and capacity. Longitudinal studies will be needed to confirm the observed associations between disease progression and physical activity and capacity among HIV-infected individuals to reduce the burden of morbidity and improve quality of life.

## Conclusions

To conclude, objectively measured levels of physical activity and capacity among HIV-infected were lower compared to HIV-uninfected individuals. Correlates which may contribute to lower levels of physical activity and capacity in HIV-infected were HIV status (CD4 count), chronic inflammation, HIV related wasting and anaemia. In contrast, for HIV-uninfected individuals, only anaemia and abdominal obesity were found to be correlated with lower physical capacity. Endurance to physical activity and changes in physical capacity depends on regular physical activity regardless of health status. Based on these findings, there is a need to improve management and treatment for HIV- infected, to develop and evaluate interventions on physical activity among HIV-infected as well as HIV-uninfected individuals to improve general health and reduce the risk of NCDs.

## Supporting information

S1 Questionnaire (DOC)

S2 Questionnaire (DOC)
