# Supplementary material for: Levels and correlates of physical activity and capacity among HIV-infected compared to HIV-uninfected individuals
Source: PLoS One. 2022 Jan 21;17(1):e0262298. doi: 10.1371/journal.pone.0262298 (PMC8782412; doi:10.1371/journal.pone.0262298)
Supplement: S1 Questionnaire — (DOC) [file pone.0262298.s001.doc]

**A: Socio-economic and demography questionnaire**

**Instructions**

***Read***: I would like to ask you some questions about yourself.

| **Q No.** | **Code** | **Questions and Filters** | **Coding Categories** | |
| --- | --- | --- | --- | --- |
| 1 | Rel | What is your religion? | None  Christian  Muslim  Hindu  (specify)…………………………………….. | 0  1  2  3  9 |
| 2 | Edlev | What is the highest level of formal education you have completed? | Never went to school  Primary school  Secondary (form one-four)  Secondary (form five-six)  Vocational or non-tertiary college  University/ tertiary college | 0  1  2  3  4  5 |
| 3 | Marst | What is your current marital status? (***Probe***) | Married/Cohabiting  Widowed  Separated/divorced  Single (never married)  Other (specify)…………………………………….. | 1  2  3  4  5 |
| 4 | Emply | Which of the following best describes your main work status in the past 12 months? | Salary employee  Self employed (fishermen)  Self employed (peasant)  Self employed (petty trader)  Self employed (other) Businessman/woman  Housewife  Unemployed  Student  Other (specify)…………………………………….. | 1  2  3  4  5  6  7  8  9 |
| 5 | Aver | On average, how much do you earn each month (from all income sources)? (***Probe so she/he gives you accurate answer*).** | TZS|__|__|__|__|__|__|__|__|  (***write 99999999 if don’t know***) |  |

Staff initials |___|___| Supervisor’s initials |___|___|

**B: Anthropometry questionnaire**

| **Q No.** | **Code** | **Activity** | **Output** |
| --- | --- | --- | --- |
| 1 | date_f | Date (dd-mmm-yyyy) | |___|___|-|___|___|___|-|___|___|___|___| |
| 2 | hght1_b  hght2_b  hght3_b | Measure and record height in cm to the nearest 0.1cm | Measurement 1 |___|___|___|.|___| cm  Measurement 2 |___|___|___|.|___| cm  Measurement 3 |___|___|___|.|___| cm |
| 3 | wght1_b  wght2_b  wght3_b | Measure and record weight in kilograms to nearest 0.1 kg | Measurement 1 |___|___|.|___| kg  Measurement 2 |___|___|.|___| kg  Measurement 3 |___|___|.|___| kg |
| 4 | waist1_b  waist2_b  waist3_b | Waist circumference (cm to nearest 0.1) | Measurement 1 |___|___|___|.|___|cm  Measurement 2 |___|___|___|.|___|cm  Measurement 3 |___|___|___|.|___| cm |
| 5 | Grip1_b  Grip2_b | Grip strength (kg to nearest 0.1) | |___|___|.|___|kg |

Staff initials |___|___| Supervisor’s initials |___|___|

**C:** Laboratory results form

| **No.** | **Codes** | **Results parameter** | **Values** |
| --- | --- | --- | --- |
| 1 | rbc | RBC (10^6^/µL) |  |
| 2 | hb | Hb (g/dL) |  |
| 3 | hct | Hct (%) |  |
| 4 | mcv | MCV (fL) |  |
| 5 | mch | MCH (pg) |  |
| 6 | Mchc | MCHC (g/dL) |  |
| 7 | rdw | RDW (%) |  |
| 8 | plt | PLT (10^3^/ µL) |  |
| 9 | mpv | MPV (fL) |  |
| 10 | wbc | WBC (10^3^/µL) |  |
| 11 | neut | Neutrophils (10^3^/µL) |  |
| 12 | lymp | Lymphocytes (10^3^/µL) |  |
| 13 | mono | Monocytes (10^3^/µL) |  |
| 14 | eosin | Eosinophils (10^3^/µL) |  |
| 15 | baso | Basophils (10^3^/µL) |  |
| 16 | cd4 | CD4 count (cells/µL) |  |
| 17 | crp | CRP (mg/L) |  |

Staff initials |___|___| Supervisor’s initials |___|___|
