# Supplementary material for: Levels and correlates of physical activity and capacity among HIV-infected compared to HIV-uninfected individuals
Source: PLoS One. 2022 Jan 21;17(1):e0262298. doi: 10.1371/journal.pone.0262298 (PMC8782412; doi:10.1371/journal.pone.0262298)
Supplement: S2 Questionnaire — (DOC) [file pone.0262298.s002.doc]

#### Socio-economic and demography questionnaire

Maelezo

Soma: Napenda kukuuliza maswahili kuhusu wewe mhusika

| **Ques** | **Code** | **Questions and Filters** | **Coding Categories** | |
| --- | --- | --- | --- | --- |
| 1 | Rel | Dini yako ni ipi? | Sina dini  Mkristo  Muislamu  Mhindu  Nyingine (taja)……………………………... | 1  2  3  4  5 |
| 2 | Edlev | Ni kipi kiwango cha juu cha elimu ulichofikia? | Sijawahi kwenda shule  Elimu ya msingi  Sekondari (kidato cha I-IV)  Sekondari (kidato cha I-VI)  Chuo cha mafunzo  Chuo cha elimu ya juu/Chuo kikuu | 1  2  3  4  5  6 |
| 3 | Marst | Hali yako ya ndoa ikoje kwa sasa? (dodosa) | Nimeoa/nimeolewa/ninaishi na mtu  Mjane/mgane  Tumetengana/talaka  Sijawahi kuoa/kuolewa  Nyingine (taja)……………………………………… | 1  2  3  4  5 |
| 4 | Emply | Katika miezi 12 iliyopita umekuwa ukijishughulisha na kazi gani? | Kazi ya mshahara  Nimejiajiri (mvuvi)  Nimejiajiri (mkulima)  Nimejiajiri (mfanyibiashara mdogo)  Nimejiajiri (mfanyi biashara mkubwa)  Mama wa nyumbani  Sina ajira  Mwanafuzi  Nyingine (taja)…………………………  Sijui/amekataa | 1  2  3  4  5  6  7  8  9  10 |
| 5 | Aver | Kwa wastani, unapata fedha kiasi gani kwa mwezi kutoka kwenye kazi zako zote? (Dodosa ili akupe jibu sahihi. Muombe ajumlishe mapato yote anayopata toka kwenye kazi zake zote) | TZS|__|__|__|__|__|__|__|__|  ***(andika 99999999 kama hajui)*** |  |

Staff initials |___|___| Supervisor’s initials |___|___|
